# “Conscious Nine Months”: Exploring Regular Physical Activity amongst Pregnant Women—A Qualitative Study Protocol

**DOI:** 10.3390/ijerph191811605

**Published:** 2022-09-15

**Authors:** Beata Makaruk, Weronika Grantham, Natalia Organista, Maciej Płaszewski

**Affiliations:** 1 Department of Sport for All, Faculty of Physical Education and Health in Biała Podlaska, Józef Piłsudski University of Physical Education, 00-968 Warsaw, Poland; 2 Department of Humanities and Social Sciences, Faculty of Physical Education, Józef Piłsudski University of Physical Education, 00-968 Warsaw, Poland; 3 Department of Rehabilitation, Faculty of Physical Education and Health in Biała Podlaska, Józef Piłsudski University of Physical Education, 00-968 Warsaw, Poland

**Keywords:** pregnancy, physical activity, regular exercise programme, qualitative research

## Abstract

Despite a clear and convincing evidence base and strong recommendations for pregnant women to maintain regular moderate physical activity throughout pregnancy, many of them reduce or discontinue exercise altogether. This is due to pregnancy-related difficulties and barriers. The aim of this protocol is to describe a qualitative research methodology for a study exploring the experiences of women who managed to achieve the recommended levels of physical activity throughout their pregnancy by regularly participating in a specially designed “Conscious nine months” exercise programme. A qualitative descriptive design will be used, including semi-structured in-depth literature-based interviews, together with thematic analysis. Consolidated criteria for reporting qualitative research (COREQ) guidelines will be used. In-depth individual interviews (60–90 min) with exercise programme participants, used together with a thematic analysis process, will allow for a better understanding and exploration of what enabled the participants to achieve such high adherence to the overall exercise programme. The chosen methodology offers a structured way for researchers to explore the experiences and factors that influence the ability of pregnant women to be physically active, enabling research into how pregnant women can be supported to remain active during this special, often challenging time in life.

## 1. Introduction

Research shows that physical activity (PA) has a beneficial effect on the physical health [1,2] and psychological well-being [3,4,5,6] of pregnant women. 

Well-balanced maternal PA has positive effects on foetal health [7]. The World Health Organization [8] recommends 150 min per week of moderate PA, 75 min of vigorous PA or a combination of both. Similarly, The American College of Obstetricians and Gynecologists recommends 150 min of moderate-intensity PA spread throughout the week [9]. A comparison of PA in pregnancy guidelines from different countries shows that most guidelines support moderate-intensity PA with varying frequency and duration [10]. 

However, despite a clear and convincing evidence base and strong recommendations, including the WHO guidance indicating the many benefits of PA, many women decrease their PA during pregnancy or refrain from exercising altogether. Evenson et al. [10], comparing studies from different countries, concluded that far too many pregnant women do not meet the required PA levels. Flannery et al. [11] also showed that only 4.7–21.5% of pregnant women, with data varying depending on the country, maintain the recommended levels of PA. In the U.S., the prevalence of women who met the guidelines for PA ranged from 12.7 to 45.0% [12].

In previous decades, a number of studies analysed both barriers and enablers for staying physically active during pregnancy [11,13,14]. The identified barriers were connected to many various factors—personal, social, and environmental—that can act as obstacles to PA [13]. Harrison et al. [15] presented pregnancy-related discomforts, fatigue, and lack of time as the main barriers, including them as intrapersonal factors. The study by Evenson et al. [14] similarly showed that the barriers identified by the participants most often concerned the intrapersonal level. The literature review of quantitative and qualitative evidence performed by Coll et al. [13] also identified several barriers, such as pregnancy-related health issues and limitations, time constraints, or perceptions of already being active during everyday tasks, but also pointed toward a lack of motivation for some women, safety concerns (mother or foetus), lack of social support, lack of advice and information, and environmental, organisational, and policy barriers (e.g., weather and lack of resources).

Furthermore, accurate information from healthcare providers, education, and communication with patients were also found to be very important in enabling a healthy lifestyle during pregnancy [11,13,16,17,18]. Still, many professionals do not seem to adequately inform their patients about the benefits of engaging in PA during pregnancy [16,17,19,20]. Misconceptions about the PA of expectant mothers can have a significant impact on reducing or discontinuing it altogether [21]. Moreover, some women are still concerned that PA during pregnancy may be dangerous to the foetus [22,23,24].

However, it is not only the increased level of knowledge and attitudes or beliefs that are important. Another study found that socio-ecological environments can also have a greater impact on lifestyle behavioural changes in pregnant women than professional health counselling [25].

On the other hand, research also shows that the factors that can support women in maintaining appropriate PA levels during pregnancy include being physically active before pregnancy, knowledge about the benefits of PA on women’s body and the foetus, personal goal setting, and the desire to feel better, as well as social support from family members (especially form partners/husbands) [11,15,26].

The complexity of factors influencing the ultimate behaviours of pregnant women indicates the need for research using a variety of methods, including qualitative methods. As Wagnild and Pollard stated, approaching PA only as a “health behaviour” “can result in an oversimplified and decontextualised understanding of how PA is (or is not) experienced and integrated into everyday life” [22] (p. 1072). Thus, qualitative methods can help to grasp the complexity of everyday behaviours and not limit them to a simple decision-making process. In-depth interviews seem to be the most appropriate way to collect the subjective experiences and “truths” of informants and will therefore be used in this study.

### 1.1. Background

Most of the available studies used interviews, focus groups, and questionnaires with pregnant women to explore their beliefs and attitudes toward PA during pregnancy [3,11,13,14,15,21,27]. They focused on women with varying experiences and levels of PA. However, what is currently missing in the literature is an examination of the perspectives of women who actually managed to maintain the recommended PA levels during their pregnancies and exploring what factors enabled them to do so.

In the years 2017–2019, around 30 women took part in the specially designed regular PA programme “Conscious nine months”. The authors of the programme noticed that the vast majority of women who started their participation were able to maintain the recommended PA levels and to continue to exercise until birth (some even postpartum). This gave rise to questions about what actually enabled them to do so, as adherence to PA in this population seems problematic, as described in the earlier-mentioned studies. 

### 1.2. Research Question

The aim of the planned study is to explore and understand the experiences of women who managed to fulfil the recommended levels of PA by participating in a regular PA programme for pregnant women. Harrison et al. [15] suggested that creating pregnancy-specific programmes is one of the important enablers of staying physically active. This may support women with pregnancy-related difficulties and provide them with the extra support they need.

Therefore, the main research question is: “What was the experience of pregnant women who regularly engaged in physical activity and who completed a specially designed exercise programme ‘Conscious nine months’”?

The authors’ aim is to gain in-depth knowledge regarding the opinions and beliefs of the studied women and to describe the contexts that facilitated their participation in the programme. They would like to explore which elements were key in enabling the women to maintain regular PA, i.e., what was perceived as most helpful, motivating, meaningful, and/or attractive to them, and, possibly, if there were any other factors that they believed to be influential. Additionally, the authors would also like to discover potential changes the women would have liked to have made to further support their participation in regular PA activities.

## 2. Materials and Methods

### 2.1. “Conscious Nine Months” PA Programme for Pregnant Women

The programme, described here to enable others to potentially repeat the procedure, was designed to be comprehensive, supervised, and continuous. The pregnant participants were training at a moderate intensity (HR of 100–145 beats per minute) twice weekly (Tuesday and Thursday) for 60–90 min, depending on the stage of pregnancy. Women who were in the later stages of pregnancy had longer sessions, with more emphasis on relaxation. The sessions included a warm-up; the main part, with strengthening exercises, elements of Pilates, antithrombotic exercises, pelvic floor exercises, and stretching; and a cool-down (breathing exercises and relaxation). Each training session included a maximum of 4–6 participants and was led by a qualified prenatal PA specialist with 12 years of experience (who was also the author of the programme and is the lead author of this work). The utmost care was taken to treat all women in an individualised manner, taking into consideration their pregnancy-related needs, as well as to create a meaningful trainer–participant relationship. The women began their participation in the programme in weeks 12–16 of their pregnancy, after obtaining written consent from their gynaecologist/obstetrician, and then regularly participated in the classes until birth (weeks 38–40 of gestation). Some of them continued postpartum. 

The programme described here was also used in a separate study, designed to evaluate and ensure foetal safety [24]. The only difference between these studies was in the frequency of participation. In the above-cited study, pregnant women exercised three times a week, whilst the women described here exercised twice a week.

### 2.2. Study Design

A descriptive qualitative research design will be used. In relation to the nuanced picture of women’s experiences towards PA during pregnancy that qualitative research makes possible, this study will utilise individual in-depth interviews as the most suitable choice. Specifically, in-depth, individual, semi-structured interviews are planned to be conducted, as well as thematic analysis (TA) to address the study’s aims.

**Figure 1:**
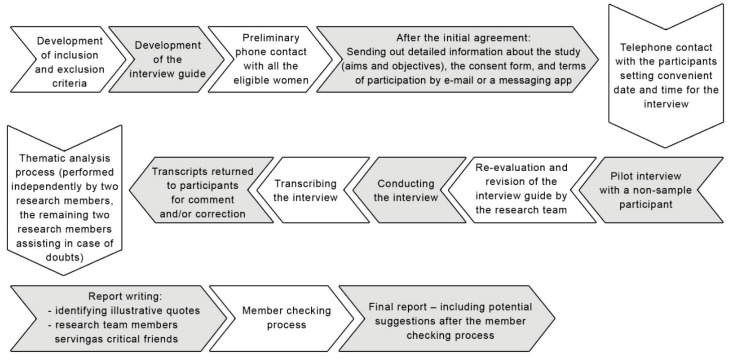
Timeline of the planned study.

This study protocol adheres to the COREQ criteria for reporting qualitative research [28]. Please see Supplementary Table S1 for the COREQ Checklist. The COREQ Flow Chart will also be filled in in the final report. The timeline of the planned study is presented in Figure 1.

### 2.3. Study Participants

Purposive sampling will be used to recruit the participants for the study, which will be conducted via telephone. Eligibility criteria are as follows:

- Inclusion criteria:

Women who took part and completed the entire “Conscious nine months” programme during their pregnancy in the years 2017–2019.

- Exclusion criteria:

Women who could not participate in the programme due to medical reasons; i.e., the physiological state of pregnancy did not allow for the exercises to continue and there was no medical clearance.

Women who do not want to agree to have their interview recorded.

### 2.4. Interview Setting

The study will be conducted in a meeting room at the Józef Piłsudski University of Physical Education in Warsaw, Faculty of Physical Education and Health in Biała Podlaska. Due to its off-campus location, this room creates comfortable conditions for the interviews, allowing for privacy and a relaxed atmosphere.

### 2.5. Recruitment Strategies and Data Collection Methods

Firstly, all eligible women will be contacted via telephone to invite them to take part. This will be followed by sending them more detailed information about the study (aims and objectives), a consent form that will need to be signed during the meeting, and terms of participation via e-mail or a messaging app so that the women can make an informed choice. Following the initial agreement, each participant will be contacted to set a convenient date and time to meet. 

Data collection via in-depth semi-structured interviews is planned to be carried out in person. However, in case of pandemic restraints, it would also be possible to perform the interviews virtually via videoconferences.

Each interview will be audio-recorded (dictaphone and telephone) and transcribed verbatim. Transcripts will then be returned to the participants for comment and/or correction.

To minimise bias, the interviews will not be conducted by the first author, who was also the women’s trainer at the time. The main interviewer is a qualified psychologist, a female, experienced in working with pregnant women, and instructed in the qualitative research approach. She has experience in communication and listening skills, and she is Polish—the same as all of the participants. The interviewer will maintain a sensitive and open-ended manner of questioning. Importantly, women will also be reassured that the researchers are interested in their authentic experiences, feelings, and views, and there are no “correct” answers to the questions.

At the conclusion of each interview, participants will be asked if they would like to add anything else about their experiences. The member checking procedure will also be used [29] to ensure that what is written in the final report depicts the women’s actual views and experiences.

The final report will also include main themes and illustrative quotes (see Supplementary Table S2).

A separate interview guide has been developed for the study using the available literature and the practical experience of the lead author and a pregnancy exercise specialist (see Supplementary File S1). The questions are mostly open-ended and focus on different subject areas. Each question also contains supplementary questions visible to the interviewer only, in case a participant requires more prompting or explanations when replying. The script will serve as a guide only. It will be the participants who will have the deciding voice as to the direction of each interview.

The interview guide will be pilot tested with a non-study sample, who will not participate in the actual study but who present characteristics corresponding to the study participants. The pilot testing will be conducted in order to improve the accuracy of data collection [29]. Following the pilot interview session, the interview guide will then be re-evaluated and reviewed by the research team to determine if any changes are needed.

Each interview will begin with some preliminary questions aimed at building a relationship with the interviewee and then move on to the individual in-depth interview based on open questions included in the prepared interview guide. At the end of each interview, participants will be asked to fill in a data sheet addressing sociodemographic information (see Supplementary File S1).

Additionally, field notes will be taken in order to record observations of the overall attitude of the interviewee during the interview. This will contain elements such as non-verbal aspects, bodily and facial expressions, gestures, speech alterations (for instance, changes in tone or volume of voice), and emotional manifestations (for instance, laughter or crying).

Interviews with 15–20 women are planned, or until theoretical data saturation is achieved [30], i.e., until data are sufficiently coherent and no new data emerge to meet the study’s objectives. Each interview is planned to last for 60–90 min.

Interviews are anticipated to begin in September/October 2022 and to be completed by December 2022.

### 2.6. Data Analysis

Data will be analysed and interpreted manually using a thematic analysis (TA) approach, which can “(…) be applied flexibly across the spectrum of ontological and epistemological positions” [31] (p. 192). In TA, themes and patterns within the transcribed data will be identified, analysed, and then reported. The process of TA will be implemented in a reflexive and recursive manner, going back and forth through the six main stages of the process, namely: familiarisation with the data, coding, theme development, theme refinement, theme naming, and writing up [32]. Thus, TA will be an active process involving not only the content of the data but also, importantly, theoretical assumptions, present knowledge about PA in pregnancy, research skills and experience of the research team, and practical experiences of the lead author.

To be more specific, coding will not be performed using an anecdotal approach but will be conducted in a thorough and comprehensive way, where themes will be checked and re-checked against each other and against the original data, in order to maintain the quality and scientific rigour of the analysis [33].

The data analysis will be performed by two research team members independently (B.M. and W.G.). There will also be discussions regarding the categorisation process amongst the whole research team, especially in case some questions or uncertainties arise.

### 2.7. Rigour

Due to qualitative research offering many methodological possibilities and approaches, judging the rigour, validity, and trustworthiness of qualitative studies is a complex process [29]. The two main competing approaches in the field of qualitative sport and exercise research are the *criteriological* approach based upon the work of Lincoln and Guba [34] and the *relativist* approach [35] (p. 334). The former assumes that all (often very different from each other) types of qualitative studies can be judged against a set of certain criteria in order to assess all aspects of methodological rigour. However, as Burke [29] pointed out, in the world of research in which reality is seen as subjective and multiple and the forms of research are so diverse, it is not appropriate to use set universal external criteria to judge scientific rigour and validity. 

Therefore, the relativist approach will be followed in this work, where the quality of a study “is both *revealed* and *resides* in the research report” [29] (p. 335), meaning that the quality of the final paper will depend not only on the thoroughness of the researchers’ work but also on how it will be received and judged by the readers and what their response will be. The criteria for ensuring the trustworthiness of the planned study will be checked and consulted after its completion, when the final report is written. However, the main criteria that can be applied for the planned study at this moment in time are presented in Table 1 and are based on the list compiled by Smith and Caddick [36] (p. 70f).

To ensure the clarity, comprehensiveness, transparency, and completeness of the report, the COREQ Checklist for reporting qualitative research (see Supplementary Table S1) and COREQ Flow Chart will be used in the final report.

### 2.8. Ethical Considerations 

This project received the consent of the Senate Ethics Committee, No. SKE-2/2022, of the Józef Piłsudski University of Physical Education in Warsaw and will also follow the Declaration of Helsinki.

During the interviews, written consent will be given by each interviewee after being reminded of the aims and objectives of the study, and they will be informed that they can withdraw at any time, without giving reasons for doing so. They will also be assured that their confidentiality will be maintained at all times and that no sensitive or identity-related information will be included in the transcripts. Furthermore, all data files will be stored securely in locked file cabinets in the University building, and all electronic files will be password-protected.

If the emotional state of a participant is perceived to be negatively affected during the interview, the interviewer will interrupt or stop the conversation to ensure that the well-being of the participant is prioritised.

## 3. Discussion

### 3.1. Strengths

It is hoped that the planned study can deepen our understanding of what enables and supports women in maintaining the recommended levels of PA throughout pregnancy. In the planned study, this will be achieved by interviewing respondents who regularly participated in the “Conscious nine months” programme. The authors wish to discover and explore the real needs of pregnant women regarding support in maintaining regular PA during this special and often challenging time. The perspective of women who managed to exercise regularly during pregnancy is needed in the subsequent development of effective training programmes or guidelines to increase their chance of success and their uptake by women, as well as to provide information on how to support pregnant women more effectively. 

So far, most studies have focused on barriers or enablers and were based on interviews with women concerning their perceptions and views on PA in general [3,11,13,14,15,21,27]. To the best of our knowledge, this is the first study that will interview women who were actually successful in completing recommended PA levels throughout their pregnancy and explore this success in depth. Moreover, previous research was carried out in Western Europe or North America, while this study focuses on Central/Eastern European women and may identify other contextual and cultural factors that influence participation and success.

### 3.2. Limitations

With regard to the limitations of the planned study, the authors acknowledge that the findings will be bound by contexts (place and time) and will be representative of a small sample of respondents. However, the validity of the study will not be compromised, and the result will depict the views of the participants who experienced being part of the regular PA programme for pregnant women.

The other limitation of the planned study also rests in not taking into consideration the views of women who, for a variety of different reasons, did not take part in the exercise programme. As known from other studies [3,11,13,14,15,21,27], factors such as lack of time, difficulties with transport to the exercise venue, or lack of childcare can be decisive in not being able to participate. However, these barriers are already widely researched. Hence, focusing on women who exercised regularly during their pregnancy might bring new insights and knowledge as to what actually helped and supported them in this process.

Another potential limitation is that the women who did not manage to participate in the exercise programme will not be included in this study. This is due to the fact that it is not possible to reach women who possibly thought about taking part but did not decide to do so. According to current research, there may be various reasons for women not to be able to take part in such programmes, including but not limited to transport difficulties, lack of time, lack of childcare, or lack of social support [3,10,11,13,15,21,27]. By interviewing women who managed to participate regularly, however, it is still possible to explore in more detail the factors that supported them in their completion of the PA programme. It should also be noted that some women who wished to participate in the programme could not do so due to medical reasons. They had certain contraindications to exercise and were not permitted to participate by their doctor.

Finally, the planned interviews will focus on women’s participation in a programme that was conducted over 3 years ago. This retrospective view might also be seen as a limitation, as it might be more difficult for women to recall certain details, or perhaps they may give less accurate reports. This is primarily connected to the logistical difficulties of contacting people during the recent pandemic. Moreover, the time delay in asking participants questions about their experiences might also result in more reflective and composed accounts. As pointed out by Willig [37], in subjective research, the most important aspect is how people perceive their reality rather than its objectivity.

## 4. Conclusions

The planned study will explore and analyse factors that enabled participants to maintain regular PA levels by taking part in the specially designed exercise programme for pregnant women, “Conscious nine months”. This will be conducted using a qualitative research approach, specifically in-depth individual interviews and TA. The results will shed light on what aspects of physical activity programmes for pregnant women might be especially meaningful to them, enabling them to maintain the recommended PA levels, taking into consideration contextual, intrapersonal, social, and environmental dimensions. This could prove vital in the future design and implementation of successful PA programmes, as well as assist in education regarding the PA and support of women during pregnancy.

## Figures and Tables

**Table 1:** Criteria for judging qualitative research.

Criterion	Description
**Substantive contribution**	Does the article contribute to the broader understanding of the studied subject, demonstrating a scientific perspective that can also be seen within the construction of the text?
**Impact**	Does the research affect the reader and generate new questions or awaken a response in a reader, e.g., for action—to try new practices or to write?
**Width**	Is the evidence comprehensive in both the conduct of the interviews and the depth of interpretation and analysis? Does the article contain quotations from participants and potential alternative explanations?
**Coherence**	Does the study seem internally complete and externally coherent with existing research/theories?
**Credibility**	The participants’ reflections will be sought on interpretations of the data; i.e., member checks will be performed in order to deepen analysis and ensure fairness and believability of interpretations.
**Transparency**	Co-authors—N. O. and M. P., serving as critical friends, scrutinising theoretical preferences, and conducting data collection and the analysis process. As they come from different subject areas other than pregnancy exercise, they can aid in uncovering potential assumptions and taken-for-granted beliefs.

## Data Availability

Not applicable.

## Supplementary Materials

The following supporting information can be downloaded at: https://www.mdpi.com/article/10.3390/ijerph191811605/s1. Supplementary Table S1: Consolidated criteria for reporting qualitative studies (COREQ): a 32-item; Supplementary Table S2: Themes and illustrative quotes; Supplementary File S1: Interview guide for the individual in-depth interviews with women who completed the “Conscious nine months” regular PA programme.
