# Supplementary material for: “Conscious Nine Months”: Exploring Regular Physical Activity amongst Pregnant Women—A Qualitative Study Protocol
Source: Int J Environ Res Public Health. 2022 Sep 15;19(18):11605. doi: 10.3390/ijerph191811605 (PMC9517471; doi:10.3390/ijerph191811605)
Supplement: Supplementary file 1 [file ijerph-19-11605-s001.zip › Supplementary File S1.pdf]

Supplementary File S1: Interview guide for the individual in-depth interviews with women who completed the "Conscious nine months" regular PA programme.

Date:

Location of the interview:

Starting time of the interview:

Ending time of the interview:

Opening questions:

Tell me a little bit about yourself, please. E.g. How many children do you have?; Do you work?; What type of work do you do and how many hours?; etc.

The main questions are written in italics. The bulleted questions are supporting or prompting questions and will only be used if a participant needs more guidance in answering them.

Area	Main questions	Supplementary questions/ prompts
**Physical skills area**	*1. Tell me about your activities before you became pregnant.*	— How active have you been?<br>— What types of activities were these?<br>— How often have you exercised/been active?<br>— Have you attended any exercise classes? If so, were those group or individual activities?<br>— What type of activity was it?<br>— What was your motivation to be active?<br>— Tell us briefly about your sporting past (sports played, was your family involved in any sports?)
	2. Could you tell me a little bit about whether you were aware of the benefits of physical activity during pregnancy?	— Has anyone told you about it/have you talked with anyone about it?<br>— Has your doctor (or other health professionals) talked to you about undertaking any physical activity? If yes, what

		recommendations or feedback have you received from your doctor on this subject?
	3. Did you think about taking up physical activity after you became pregnant? What prompted you to take up such an activity?	
	4. Was there any difficulties or challenges for you to start exercising while pregnant?	— Time<br>— Access to information on the impact of physical activity on the health of the pregnant woman and her baby<br>— Well-being (physical, mental, emotional) during pregnancy<br>— Other health problems/ complications<br>— Social support (family, partner/husband)<br>— Opinions of those around you about being active during pregnancy
	5. What made you decide to take part in the "Conscious nine months" physical activity programme for pregnant women?	— How did you find out about the programme of physical activity for pregnant women "Conscious nine months"?<br>— Why these classes and not others?
	6. How did you feel before starting the programme?	— Expectations<br>— Any concerns
**Experience of participating in the programme**	*7. Please tell us what was your experience of participating in the* "Conscious nine months" *programme?*	— What was important to you?<br>— What do you remember most?
	8. What do you think you found most helpful about participating in the programme?	
	9. Was there any difficulties you encountered during your participation?	— Did anything bother you?<br>— Did you have any concerns?
	10. Do you think that your participation in this exercise	

	programme had any impact on your relationship with your baby?	
**Physical area**	*11. How did you feel during the sessions?*	— Safety (what did the measurements before/during/after the class mean to you)<br>— The attractiveness of the sessions<br>— Atmosphere<br>— Level of difficulty of exercises<br>— Selection of exercise types
	12. Could you tell me more about the effect that your participation in the exercise programme had on your well-being?	— Energy level<br>— Sleep<br>— Ailments related to pregnancy<br>— Emotional state
	13. Do you think that taking part in the programme may have had any impact on the birth of your baby?	— Pain level<br>— Level of stress<br>— Sense of preparation for childbirth
**Social area**	*14. What role did the pregnancy exercise instructor play for you throughout the entire programme?*	— Relationship<br>— Trainer's approach<br>— Way of instructing exercises<br>— Level of knowledge
	15. How important was it for you to exercise in a group of other pregnant women?	
	16. How did your loved ones approach your participation in the programme?	— Your partner/husband?<br>— Your family and your husband's/partner's family?<br>— Friends/community?<br>— Was there anyone from your environment who explicitly expressed opposition to your participation in the programme?<br>— If the respondent answers that others had a critical, negative, or reserved attitude towards her exercise - how did you deal with these types of statements?
**Knowledge area**	*17. What was the doctor's opinion on your participation in the programme during your pregnancy?*	— How did this doctor's position influence your decision to participate in the programme?

	18. Has participation in the programme had any impact on your level of knowledge regarding physical activity during pregnancy?	
**Area of behavioural change**	*19. What helped you to persevere with physical activity throughout your entire pregnancy?*	— What factors
	20. What has influenced your motivation to exercise?	
**Environmental area**	*21. What influence did environmental factors have on your participation in the programme, such as…*	— Work<br>— Home responsibilities<br>— Available time<br>— Finances<br>— Weather<br>— Proximity to home
	22. Would you change anything in the "Conscious nine months" programme? If so, what that be?	
	23. Is there anything I haven't asked you today that you would like to mention?	

After the interview, please complete the following sociodemographic data:

Year of birth:

Place of residence:

Nationality:

Marital status:

Education:

Occupation:

Place of work:

The following questions refer to the period when you participated in the exercise programme:

At approximately which week of pregnancy did you stop working?

Have you been on sick leave while pregnant?

If yes, from which week of pregnancy were you on sick leave?

Was it your first pregnancy? If not, which one?

At what week of pregnancy did you start taking part in the exercise programme?
