# Supplementary material for: “Conscious Nine Months”: Exploring Regular Physical Activity amongst Pregnant Women—A Qualitative Study Protocol
Source: Int J Environ Res Public Health. 2022 Sep 15;19(18):11605. doi: 10.3390/ijerph191811605 (PMC9517471; doi:10.3390/ijerph191811605)
Supplement: Supplementary file 1 [file ijerph-19-11605-s001.zip › Supplementary Table S1.pdf]

Supplementary Table S1: Consolidated criteria for reporting qualitative studies (COREQ): a 32-item checklist.

No	Item	Guide questions/description
**Domain 1: Research team and reflexivity**		
Personal Characteristics		
1.	Interviewer/facilitator	Interviews will be conducted by a psychologist instructed in qualitative research (not a co-author of the planned study).
2.	Credentials	B.M. - PhD, W.G. - MA, N.O. – PhD, M.P. - PhD
3.	Occupation	B.M. – lecturer, researcher, pregnancy exercise specialist; W.G. – lecturer, researcher, yoga and somatic movement practitioner; N.O. – lecturer, researcher; M.P. – lecturer, researcher;
4.	Gender	B.M., W.G., N.O. – females, M.P. – male;
5.	Experience and training	What experience or training did the researcher have?<br>B.M. – Eight years experience in pregnancy-related research, thirteen years experience as a pregnancy exercise specialist and seventeen years experience as an athletics trainer; W.G – Phd candidate, seven years experience in qualitative methodology; N.O. - Seven years experience in qualitative methodology and gender studies research; university adjunct; M.P. specialist physiotherapist, university professor;
Relationship with participants		

No	Item	Guide questions/description
6.	Relationship established	The first author of the planned study has a previous relationship with the participants as an exercise trainer. The remaining research team members do not have any previous relationship with the participants.
7.	Participant knowledge of the interviewer	The participants know the first author of this work as a pregnancy exercise specialist, as they all trained with her during their pregnancy. Additionally, they know she is a lecturer and researcher, interested in the subject of pregnancy exercise and health. Hence, they know the goal and reasons for doing this research are to explore what may support women in maintaining regular physical activity during pregnancy and improve training programmes for pregnant women.
8.	Interviewer characteristics	The interviewer will be a psychologist, instructed in qualitative research. To reduce the risk of bias, the research team decided the interviews will not be conducted by the first author of the planned study, because of her relationship with the participants. Thus, a trained and experienced psychologist was chosen, who is a neutral person, a female, with experience in communication and listening skills (Polish – the same as all the participants). Moreover, the interviewer has a personal interest in health and pregnancy topics, as she also works with pregnant women in a local hospital.
**Domain 2: study design**		
Theoretical framework		
9.	Methodological orientation and Theory	Thematic Analysis (TA)

No	Item	Guide questions/description
Participant selection		
10.	Sampling	Purposive sampling.
11.	Method of approach	Telephone initially to arrange interviews. Interviews are planned to be conducted face-to-face.
12.	Sample size	15-20.
13.	Non-participation	N.A. in the protocol (this will be mentioned in the final report of the planned study).
Setting		
14.	Setting of data collection	Movement Studio belonging to the University.
15.	Presence of non-participants	No.
16.	Description of sample	Women who completed the "Conscious nine months" exercise programme during their pregnancy in the years 2017-2019.
Data collection		
17.	Interview guide	An interview guide with questions and prompts is provided as a supplementary file. It is planned to be pilot tested.
18.	Repeat interviews	N.A. in the protocol (this will be mentioned in the final report of the planned study).
19.	Audio/visual recording	The data will be audio recorded.
20.	Field notes	Field notes will be made during and/or after the interviews.

No	Item	Guide questions/description
21.	Duration	60-90 mins.
22.	Data saturation	Yes – in the main text of the protocol.
23.	Transcripts returned	The transcripts will be returned to participants for comment and/or correction.
**Domain 3: analysis and findings**z		
Data analysis		
24.	Number of data coders	Two coders working independently and two other research members will be involved in case of doubts/ problems.
25.	Description of the coding tree	N.A. in the protocol (this will be mentioned in the final report of the planned study).
26.	Derivation of themes	Themes will be derived from the data.
27.	Software	None.
28.	Participant checking	Participants will be asked to provide feedback on the findings.
Reporting		
29.	Quotations presented	Participant quotations will be presented to illustrate the themes/findings (table provided as a supplementary file). Each quotation will be identified (by the participant number).
30.	Data and findings consistent	N.A. in the protocol (this will be mentioned in the final report of the planned study).
31.	Clarity of major themes	N.A. in the protocol (this will be mentioned in the final report of the planned study).

No	Item	Guide questions/description
32.	Clarity of minor themes	N.A. in the protocol (this will be mentioned in the final report of the planned study).
