# Supplementary material for: “Conscious Nine Months”: Exploring Regular Physical Activity amongst Pregnant Women—A Qualitative Study Protocol
Source: Int J Environ Res Public Health. 2022 Sep 15;19(18):11605. doi: 10.3390/ijerph191811605 (PMC9517471; doi:10.3390/ijerph191811605)
Supplement: Supplementary file 1 [file ijerph-19-11605-s001.zip › Supplementary Table S2.pdf]

Supplementary Table S2: Themes and illustrative quotes.

Themes	Illustrative quotes
**Physical skills area (physical activity before starting the exercise programme)**	
**Experience of participating in the "Conscious 9 months" exercise programme**	
**Physical area**	
**Social area**	
**Knowledge area**	

**Behavioural changes area**	
**Environmental factors**	
**Others**	
